# 

**Published:** 1879-06

**Authors:** 


					﻿Whole Number,
CCCCIX.
June, 1879.
Number 6,
Vol. XXXVIII.
THE
CHICAGO
MEDIC AL
T ournal and Examiner
EDITORS:
WILLIAM H. BYFORD, A.M., M.D.,
JAS. NEVINS HYDE, A.M., M.D. FERD. C. HOTZ, M.D.
E. FLETCHER INCALS, M.D.
CHICAGO:
PUBLISHED BY THE CHICAGO MEDICAL PRESS ASSOCIATION.
188 Clark Street.-
^“Read the Notice of this Journal among Advertisements.
				

